# Polyethylene Glycol-Mediated Synthesis of Cubic Iron Oxide Nanoparticles with High Heating Power

**DOI:** 10.1186/s11671-015-1091-0

**Published:** 2015-10-07

**Authors:** Cristian Iacovita, Rares Stiufiuc, Teodora Radu, Adrian Florea, Gabriela Stiufiuc, Alina Dutu, Sever Mican, Romulus Tetean, Constantin M. Lucaciu

**Affiliations:** Department of Pharmaceutical Physics-Biophysics, Faculty of Pharmacy, “Iuliu Hatieganu” University of Medicine and Pharmacy, Pasteur 6, 400349 Cluj-Napoca, Romania; Interdisciplinary Research Institute on Bio-Nano-Science, Treboniu Laurian 42, 400271 Cluj-Napoca, Romania; National Institute of Research and Development for Isotopic and Molecular Technologies, Donath 65-103, 400293 Cluj-Napoca, Romania; Department of Cell and Molecular Biology, Faculty of Medicine, “Iuliu Hatieganu” University of Medicine and Pharmacy, Pasteur 6, 400349 Cluj-Napoca, Romania; Faculty of Physics, “Babes Bolyai” University, Kogalniceanu 1, 400084 Cluj-Napoca, Romania; Department of Physiology, Faculty of Medicine, “Iuliu Hatieganu” University of Medicine and Pharmacy, Clinicilor 1, 400006 Cluj-Napoca, Romania

**Keywords:** Cubic iron oxide magnetic nanoparticles, Polyethylene glycol, Magnetic hyperthermia, Specific absorption rate, Dipole-dipole interactions, Brownian friction

## Abstract

Iron oxide magnetic nanoparticles (IOMNPs) have been successfully synthesized by means of solvothermal reduction method employing polyethylene glycol (PEG200) as a solvent. The as-synthesized IOMNPs are poly-dispersed, highly crystalline, and exhibit a cubic shape. The size of IOMNPs is strongly dependent on the reaction time and the ration between the amount of magnetic precursor and PEG200 used in the synthesis method. At low magnetic precursor/PEG200 ratio, the cubic IOMNPs coexist with polyhedral IOMNPs. The structure and morphology of the IOMNPs were thoroughly investigated by using a wide range of techniques: TEM, XRD, XPS, FTIR, and RAMAN. XPS analysis showed that the IOMNPs comprise a crystalline magnetite core bearing on the outer surface functional groups from PEG200 and acetate. The presence of physisorbed PEG200 on the IOMNP surface is faintly detected through FT-IR spectroscopy. The surface of IOMNPs undergoes oxidation into maghemite as proven by RAMAN spectroscopy and the occurrence of satellite peaks in the Fe2p XP spectra. The magnetic studies performed on powder show that the blocking temperature (T_B_) of IOMNPs is around 300 K displaying a coercive field in between 160 and 170 Oe. Below the T_B_, the field-cooled (FC) curves turn concave and describe a plateau indicating that strong magnetic dipole-dipole interactions are manifested in between IOMNPs. The specific absorption rate (SAR) values increase with decreasing nanoparticle concentrations for the IOMNPs dispersed in water. The SAR dependence on the applied magnetic field, studied up to magnetic field amplitude of 60 kA/m, presents a sigmoid shape with saturation values up to 1700 W/g. By dispersing the IOMNPs in PEG600 (liquid) and PEG1000 (solid), it was found that the SAR values decrease by 50 or 75 %, indicating that the Brownian friction within the solvent was the main contributor to the heating power of IOMNPs.

**Electronic supplementary material:**

The online version of this article (doi:10.1186/s11671-015-1091-0) contains supplementary material, which is available to authorized users.

## Background

In the recent decades, extensive research has been focused on the synthesis of magnetic nanoparticles (MNPs) possessing new and attractive properties (good chemical stability, low toxicity, high magnetic saturation, etc. [1]) that could propel them as ideal candidates for multiple biomedical applications such as contrast agents for magnetic resonance imaging [2], targeted drug/gene/RNA delivery [3], heat generators for magnetic hyperthermia [4], and magnetic separation and purification of biomolecular species [5]. Among all types of magnetic nanoparticles (MNPs) developed so far, the Fe_3_O_4_ are the only type of MNPs approved for clinical use by the US Food and Drug Administration [6]. By taking advantage of their heat-releasing capabilities as a result of their interaction with an external magnetic field, these MNPs have been recently used in several clinical trials for prostate and brain cancer treatment by means of magnetic hyperthermia [7, 8], a technique based on the higher heating sensitivity of cancer cells to heating as compared to healthy ones.

It is well know that when exposed to an external alternating magnetic field, the amount of heat released in the medium strongly depends on MNP-specific absorption rate (SAR), also called specific loss power (SLP) [9]. Thus, SAR (which is equal to the rate at which energy is adsorbed per nanoparticles unit mass at a specific frequency) has become one of the important parameter describing MNP heating efficiency capabilities. It has been shown that SAR can by substantially raised by increasing both the frequency (*f*) and the amplitude (*H*) of the external alternating magnetic field [9]. Based on some physiological considerations, the maximum allowed values of the two parameters are limited in biological applications, i.e., the *H* × *f* factor should not exceed a threshold value of 5 × 10^9^ Am^−1^s^−1^ [10]. For human applications, the occurrence of several side effects (e.g., tissue heating and/or muscular stimulation [11]) must be taken into account above this threshold value. SAR is also strongly dependent on various intrinsic parameters of MNPs such as their size, shape, dispersity, chemical composition, surface coating, and saturation magnetization [12, 13]. As a consequence, it is widely accepted that by carefully controlling and tuning these properties, a substantial increase of their hyperthermia performance can be achieved.

In this scientific context, a plethora of synthetic methods, with the aim of engineering MNPs with exceptional SAR values, have been developed so far [14]. The best Fe_3_O_4_ nanoparticles (NPs) in terms of SAR values have been synthesized using a non-hydrolytic thermal decomposition method [15–17]. This technique, which consists in the thermal decomposition of magnetic precursors in organic solvents, enables the engineering of monodispersed spherical or cubical Fe_3_O_4_ NPs with single crystallinity and controlled size [15–18]. Recent reports have shown that spherical Fe_3_O_4_ NPs exhibit SAR values in between 400 and 700 W/g (depending on their size) [19–21], approximately five times higher than the Feridex commercially available Fe_3_O_4_ NPs (115 W/g). Higher SAR values, up to several thousands of W/g, have been recently reported for Fe_3_O_4_ NPs exhibiting a cubic shape [22]. As the final aim is to use these Fe_3_O_4_ nanoparticles for human clinical applications, it became compulsory to transform their hydrophobic character into a biocompatible, hydrophilic one. Different strategies have been proposed for overcoming this problem, consisting mainly in coating them with biocompatible hydrophilic molecules [23, 24]. Among a wide range of biopolymers employed so far, polyethylene glycol (PEG) seems to hold a great promise [22, 25–28]. PEG is a biologically safe and degradable polymer, highly soluble in aqueous solutions preventing MNP self-aggregation and undesired adsorption of plasma proteins (opsonization) onto their surface [22]. Since PEG does not possess groups that can bind to the nanoparticle surface, it can be chemically modified by introducing active functional groups that enable its further attachment to the nanoparticle surface. Apart from the biocompatible hydrophilic character provided upon functionalization with PEG, in a recent study, it was shown that surface coating of Fe_3_O_4_ NPs with phosphorylated methoxyPEG induces a significant increase of the SAR value [27]. On the other hand, in order to avoid additional steps in the formation of hydrophilic MNPs, different synthesis approaches have been developed for the synthesis of MNPs exhibiting excellent water solubility [13]. The polyol process, based on the reduction of magnetic precursors to MNPs in liquid polyol at elevated temperatures, giving rise to monodispersed, highly crystalline, and superparamagnetic MNPs at room temperature is such an example [29, 30]. Among the most used classes of polyols one can find ethylene glycol, di-ethylene glycol, try-ethylene glycol, and tetra-ethylene glycol [29]. Even though PEG of different molecular weights has been extensively used as reducing and stabilizing agent for engineering either silver or gold nanoparticles [31, 32], its capability to produce MNPs was not tested so far.

In this study, we extend the synthesis method developed by Deng et al. [33], by replacing the ethylene glycol with PEG200 (molecular weight 200 g/mol) as solvent in a one-step synthesis method of water-soluble iron oxide magnetic nanoparticles (IOMNPs). Our approach gave rise to polydispersed F_3_O_4_ magnetic nanoparticles exhibiting cubic shape, instead of spherical shape reported by Deng et al. [33]. The average edge length can be tuned by modifying the reaction time and the amount of PEG200. The structural properties and surface composition of the IOMNPs have been thoroughly investigated by means of several complementary techniques as TEM, XRD, XPS, FT-IR, and Raman spectroscopy. The magnetic properties investigated by VSM indicate a ferromagnetic behavior of IOMNPs at room temperature pointing out a strong dipole-dipole inter-particle interaction, leading to their aggregation in water. Despite this, the heating rate of IOMNPs in water (0.5 mg/ml) at *H* × *f* factors close to biological limit reaches values between 910 and 1700 W/g.

## Methods

All the reagents employed in this study were of analytical grade and were used without any further purification. The synthesis of magnetic nanoparticles has been performed with the following products: iron(III) chloride hexahydrate (FeCl_3_ 6H_2_O) (Roth, ≥98 %), polyethylene glycol 200 (PEG200) (Roth, ≥99 %), and sodium acetate trihydrate (NaAc) (Roth, ≥99.5 %).

The general synthetic procedure for the preparation of iron oxide magnetic nanoparticles was as follows: FeCl_3_ 6H_2_O (0.675 g) and sodium acetate (NaAc) (1.8 g) were mixed and dissolved in either 60 or 90 ml of PEG200. The solutions were stirred thoroughly at room temperature for 30 min, transferred in sealed glass bottles, and heated at 240 °C for 6, 8, 10, and 12 h. The final temperature was reached at heating rates of 43 and 5 °C/min. The glass bottles were let to cool at room temperature, the excess liquid was discharged, and the obtained black precipitates were washed with ethanol, several times, in order to remove the excess of ligands and unreacted precursors. Finally, the black precipitates were dispersed and kept in double distilled water for further analysis.

TEM images were taken on a Jeol JEM 1010 transmission electron microscope (Jeol Ltd., Tokyo, Japan), equipped with a Mega VIEW III camera (Olympus, Soft Imaging System, Münster, Germany), operating at 80 kV. For TEM examination, 5-μl drops of each solution were deposited on carbon-coated copper grids. After 1 min, the excess water was removed by filter paper and the samples were left to dry under ambient air.

X-ray diffraction (XRD) measurements were carried out on powder samples at room temperature on a Bruker D8 Advance diffractometer using Cu Kα radiation. The lattice parameters and phase percentages were calculated using the FullProf software.

XPS measurements were performed with a SPECS PHOIBOS 150 MCD instrument, equipped with monochromatized Al Kα radiation (1486.69 eV) at 14 kV and 20 mA and a pressure lower than 10^−9^ mbar. The binding energy scale was charge referenced to the C1s photoelectron peak at 285 eV. A low energy electron flood gun was used for all measurements to minimize sample charging. The elemental composition on the outermost layer of samples (about 5 nm deep from surface) was estimated from the areas of the characteristic photoelectron lines in the survey spectra assuming a Shirley type background. High-resolution spectra were recorded in steps of 0.05 eV using analyzer pass energy of 30 eV. The spectra deconvolution was accomplished with Casa XPS (Casa Software Ltd., UK).

The mid-infrared spectrum of powder IOMNPs, sodium acetate, and PEG200 was recorded on a Jasco 4000 FTIR spectrometer in attenuated total reflectance (ATR) mode using a one reflection ATR accessory with ZnSe crystal. The detection system consisted in a DTGS detector, the spectral resolution of the recorded FT-IR spectrum being 4 cm^−1^.

Raman measurements were recorded using a multilaser confocal Renishaw InVia Reflex Raman spectrometer. The wavelength calibration was performed by using a silicon waver buffer. The 633-nm laser line of a He–Ne laser was employed as the excitation source. The Raman spectra were recorded on powder deposited on aluminum-covered glass, with a 50× objective and an acquisition time of 10–20 s, while the emitting laser power was varied between 15 mW and maximum value of 150 mW. The spectral resolution of the spectrometer was 0.5 cm^−1^.

Dynamic light scattering (DLS) measurements were taken using a Zetasizer Nano ZS90 (Malvern Instruments, Worcestershire, UK) in a 90° configuration. One cycle of 30 measurements was performed for each sample.

Magnetic measurements were performed on powder samples in the 4–300 K temperature range in external applied fields up to 2 T, using a vibrating sample magnetometer (VSM) produced by Cryogenic Limited.

Hyperthermia measurements were recorded with a magnetic heating system Easy Heat 0224 provided by Ambrell (Scottsville, NY, USA). The samples, usually 0.5 ml of IOMNP suspensions at different concentrations were placed in a thermally insulated vial, at the center of an 8-turn coil, connected to the remote heat station of the device. With this setup, alternating magnetic fields with strengths up to 65 kA/m and frequencies between 100 and 400 kHz were generated in the center of the coil. The temperature was measured using a fiber-optic probe, placed in the center of the vial, connected to a computer, providing the temperature values each second. The calibration of the setup, the recording protocol of temperature change versus time, and the SAR calculation are briefly described in the “Additional File”.

## Results and Discussion

### Structural Characterization of IOMNPs

**Fig. 1:**
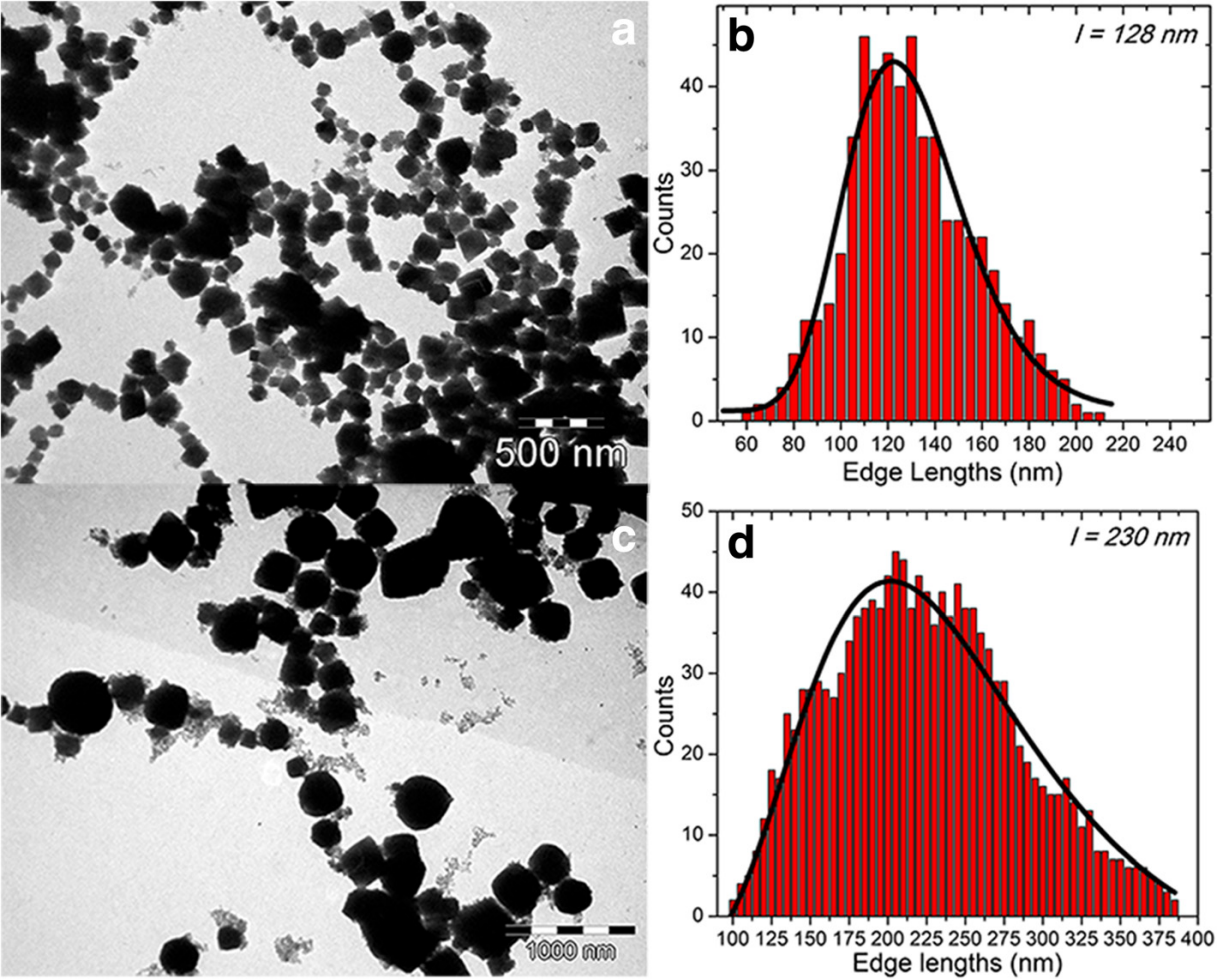
TEM images (**a**, **c**) of water-soluble cubic IOMNPs obtained in 60 ml of PEG200 for a 6- and 12-h reaction time and their corresponding size distribution histograms (**b**, **d**) fitted to a log-normal distribution (*black line*)

As it was mentioned above, polyethylene glycol (PEG) was not tested so far as a reducing agent in the synthesis of MNPs. In this paper, the reduction reaction of FeCl_3_ by PEG200 in the presence of sodium acetate, in a solvothermal system, has been performed for several magnetic precursors/PEG200 ratios. In this regard, we have fixed the amount of magnetic precursor to 0.675 g while the volume of PEG200 was gradually increased from 10 to 120 ml. It was observed that for PEG200 volumes lower than 60 ml, the IOMNPs cannot be synthesized. The formation of IOMNPs starts for 60 ml of PEG200. TEM size analysis indicates that IOMNPs, synthesized in 60 ml of PEG200 at 240 °C for 6 h, have a cubic shape (Fig. 1a) and are poly-dispersed with a broad edge length distribution (Fig. 1b); the mean edge length being 128 nm. The cubic shape of the IOMNPs is preserved if the temperature rate used to reach the final reaction temperature of 240 °C was diminished to 5 °C/min (Additional file 1: Figure S1a). In this case, the mean edge length of the cubic IOMNPs increases to 139 nm (Additional file 1: Figure S1b). Based on previously reported results [26, 34], it is believed that a low heating rate promotes nucleation at lower temperatures thus enabling the formation of a few seeds. Once formed, the seeds will further prevent further nucleation at high temperatures by quickly consuming monomers and thus developing larger nanoparticles. The cubic edge lengths of the poly-dispersed IOMNPs are further increased as the reaction time at 240 °C is longer than 6 h. No important changes were observed for reaction time of 8 or 10 h. A significant increase in the mean cubic edge lengths of the poly-dispersed cubic IOMNPs to 230 nm was observed starting with a reaction time of 12 h (Fig. 1c, d).

**Fig. 2:**
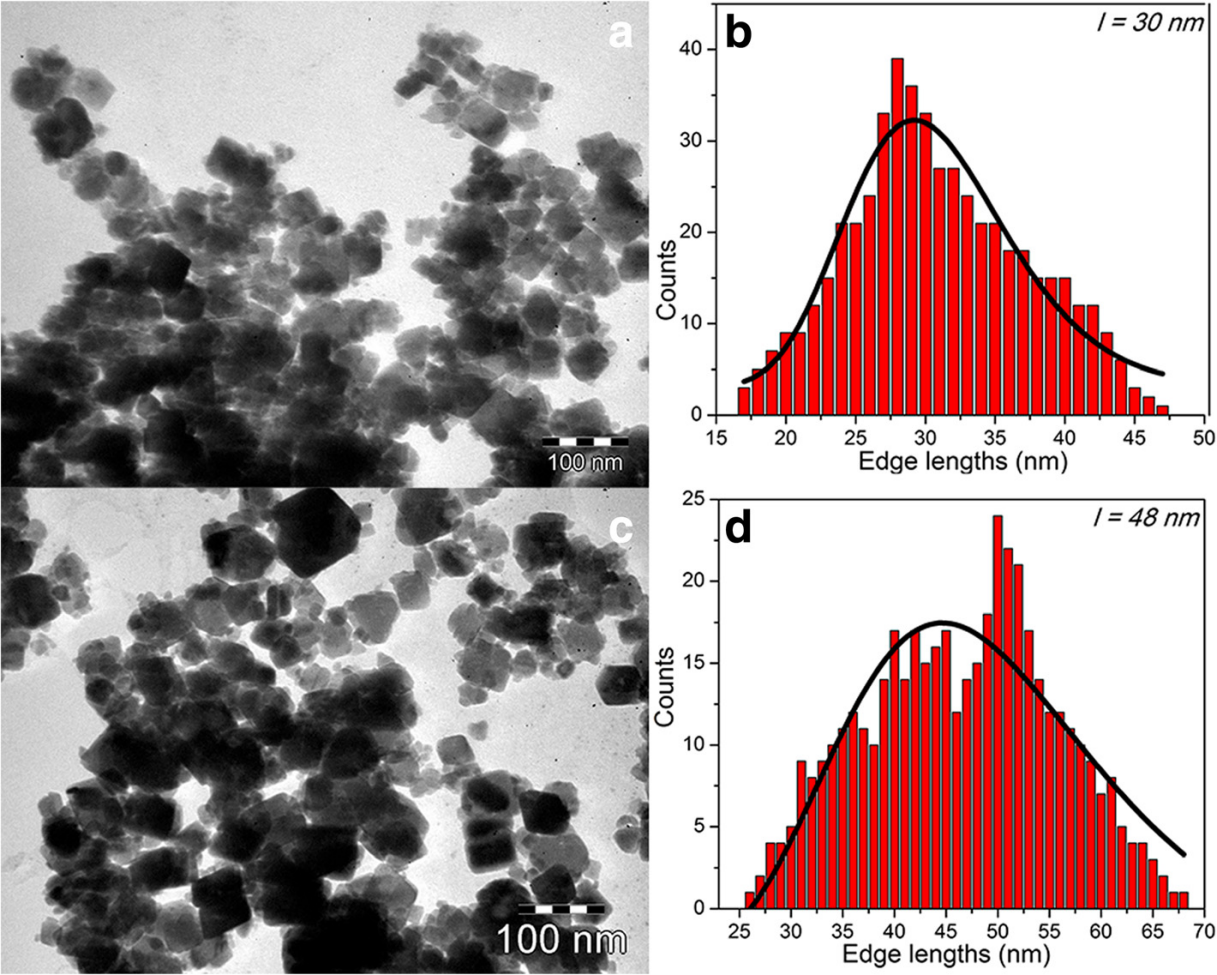
TEM images (**a**, **c**) of water-soluble cubic and polyhedral IOMNPs obtained in 90 ml of PEG200 for 6- and 12-h reaction time and their corresponding size distribution histograms (**b**, **d**) fitted to a log-normal distribution (*black line*)

Furthermore, the PEG200 volume was increased to 90 ml while keeping unchanged the reaction condition. In this case, the TEM images exhibited the coexistence of poly-dispersed cubic and polyhedral IOMNPs (Fig. 2a, c) with lower dimensions. As expected, the size of IOMNPs gradually increased with the reaction time. For instance, for a reaction time of 6 h at 240 °C, the IOMNPs have a mean size of 30 nm, whereas for 12 h, the mean size increases around 48 nm (Fig. 2b, d). No significant changes in the morphology and size were observed when the PEG200 volume was further increased to 120 ml. It is worth noting that we could not increase the reaction temperatures above 240 °C as PEG200 starts to boil. Also, below 240 °C, the nanoparticle synthesis cannot be performed. At the same time, for a reaction temperature of 240 °C, the synthesis can be completed only for reaction times longer than 6 h. Consequently, the formation of IOMNPs in the proposed solvothermal system, employing PEG200 as reducing agent, requires at least 60 ml of PEG200, a temperature of 240 °C, and a reaction time of minimum 6 h. The samples were also analyzed by DLS and the hydrodynamic diameters of the IOMNPs were found between 400 and 800 nm, indicating that IOMNPs aggregate in aqueous solutions (Additional file 1: Figure S7 and Table S7), although the samples were treated with tetramethylammonium hydroxide (TMAOH) as discussed below. Thus, the application of DLS for further characterizing both the size and shape of IOMNPs is limited.

The water solubility of cubic IOMNPs synthesized by means of laborious non-hydrolytic methods can be achieved through an additional step based on ligand exchange reaction as it was previously reported [18, 22, 25, 26]. The herein presented procedure allows—in a one-step process—the synthesis of water-soluble cubic IOMNPs in a very rapid and facile manner using PEG200 as reducing agent. Moreover, by taking into account that polyols used in the polyol-mediated synthesis enabled the preparation of high-quality water stable IOMNPs having a spherical shape [29, 30, 33], one can suppose that PEG200 acts also as a shape-directing agent, thus giving rise to either cubic or polyhedral IOMNPs. The variation of IOMNP shape upon the increase of the amount of PEG200 may rely on the ratio of magnetic precursor/surfactant. Since the selective adsorption of surfactant on a particular surface is dependent on surface energy [35] and the high-index crystallography planes possess higher surface energy, the IOMNPs will tend to be surrounded by low-index planes, such as the {111}, {110}, and {100} planes. Based on previous studies on the growth mechanism of different MNPs in the presence of oleic acid [36–38], it can be speculated that PEG200 will tend to accumulate on the (110) surface, inducing a faster growth in the <111> direction and thus leading to the formation of cubic IOMNPs. When the amount of PEG200 is increased, there will be a competing growth in both the <111> and <100> directions, resulting in the formation of polyhedral IOMNPs [39].

**Fig. 3:**
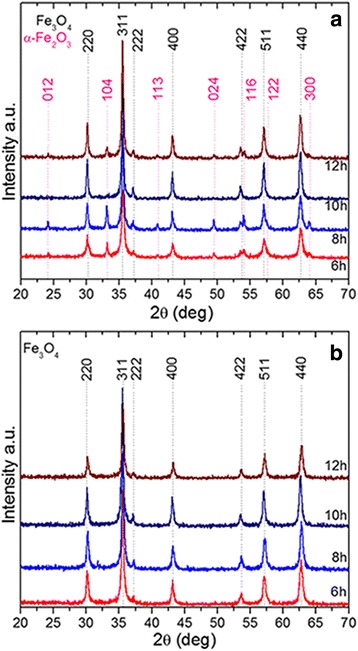
X-ray powder diffraction patterns of IOMNPs prepared in 60 (**a**) and 90 ml (**b**) of PEG200

Typical XRD patterns of IOMNPs synthesized in either 60 of 90 ml of PEG200 for 6, 8, 10, and 12 h taken at room temperature are presented in Fig. 3a, b. In the case of IOMNPs prepared in 60 ml of PEG200, the XRD patterns reveal the coexistence of two phases. The position and the relative intensities of the diffraction peaks ascribe the two phases to magnetite Fe_3_O_4_ (PDF number: 88-0315 [40]) and hematite α-Fe_2_O_3_ (PDF number: 89-0596 [41]). Since the mean size of the nanoparticles is greater than 100 nm, all diffraction peaks existing in the XRD patters are sharp. The corresponding lattice parameters for the four samples are listed in Additional file 1: Table S1 and are very closed to those of bulk samples (*a* = 8.375 Å for Fe_3_O_4_ and *a* = 5.037 Å, *c* = 13.771 Å for α-Fe_2_O_3_). In Additional file 1: Table S1, the percentage concentrations of both crystalline phases (Fe_3_O_4_ and α-Fe_2_O_3_) are calculated based on our experimental XRD data. It can be observed that the concentration of hematite is very low compared with that of magnetite and varies randomly as a function of the reaction time.

The nanoparticles prepared in 90 ml of PEG200 showed an improved crystallinity as it was demonstrated by the position and the relative intensities of their diffraction peaks, matching well with the standard XRD data for bulk magnetite (PDF number: 88-0315 [40]). Moreover, no peaks of any other phases are observed, indicating the high purity of the products. The black color of the powders can be considered a further confirmation of the fact that samples contain mainly magnetite phase and not maghemite (brown), possessing the same spinel structure and very similar XRD pattern [42]. The lattice parameters for these samples (listed in the Additional file 1: Table S2) are close to those of bulk material. The formation of an additional hematite phase for the samples synthesized using 60 ml of PEG200 may be explained by the transformation of a part of the spinel structured iron oxide under oxidative conditions. It has been observed that the addition of PEG200 in a larger quantity (synthesis performed using 90 ml of PEG200) prevents the formation of the hematite phase and reduces the oxidation degree of the spinel structure, as it was previously reported [43, 44].

### The Surface Composition of IOMNPs

**Fig. 4:**
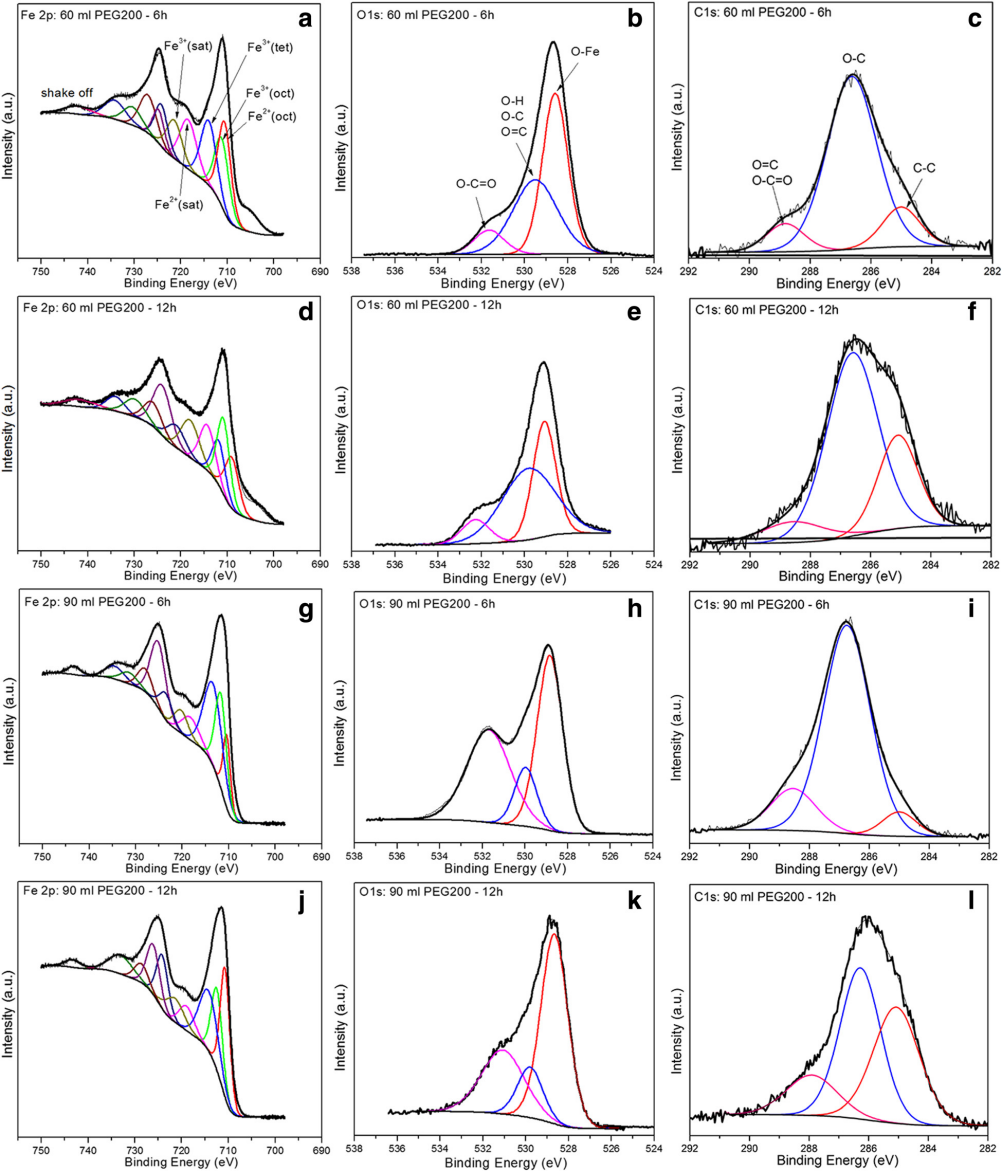
Fe2p (*left panels*; **a**, **d**, **g**, **j**), O1s (*middle panels*; **b**, **e**, **h**, **k**), and C1s (*right panels*; **c**, **f**, **i**, **l**) XP spectra of the investigated samples

X-ray photoelectron spectroscopy (XPS) is a surface-sensitive technique, probing the outermost 5–10 nm of the MNPs. Figure 4 shows the Fe2p, O1s, and C1s XP spectra of the IOMNPs synthesized in 60 and 90 ml of PEG200. The Fe2p XPS spectra of all analyzed samples are similar with that of macro-scaled crystalline Fe_3_O_4_ (Fig. 4a, d, g, j). The Fe2p spectra have been deconvoluted according to the procedure presented in [45], and consequently each region (Fe2p_1/2_ and Fe2p_3/2_) can be deconvoluted to the sum of five peaks. For the Fe2p_3/2_ region, the lowest binding energy peak at 709.4 eV is attributed to Fe2^+^ octahedral species, which has a corresponding satellite at 714.4 eV. The Fe3^+^ octahedral and tetrahedral species are found at binding energies of 710.5 and 711.8 eV, while their satellite is located at 718.6 eV. According to [45] and [46], very small MNPs (around 10 nm) induce a weak satellite structure in the XPS spectra. In our case, the observed satellite peaks are more intense than those reported in previous studies [45, 46] and the Fe2^+^ and Fe3^+^ shake-up features can be easily resolved, proving that IOMNPs size is larger than 100 nm as it was further confirmed by our TEM and XRD analysis. For the samples prepared in 90 ml of PEG200, the satellite peaks are significantly reduced (Fig. 4g, j), indicating that the corresponding Fe_3_O_4_ MNPs exhibit a smaller size compared to their counterparts prepared in 60 ml of PEG200. This observation is also reasonably supported by the TEM images. The peak located at very low binding energy (704.6 eV) is an instrumental artifact. The Fe2^+^/Fe3^+^ ratio for samples prepared in either 60 or 90 ml of PEG200 (see Additional file 1: Table S3) is close to the predicted value of 0.5 expected from the stoichiometry of Fe_3_O_4_. Nevertheless, the satellite peaks in the Fe2p XPS spectra of all samples suggest that the surface of IOMNPs is partially oxidized.

In order to clarify this issue, a detailed analysis of the O1s and C1s spectra was accomplished. The deconvolution of the O1s and C1s XPS regions provide information related to the chemical environment changes of the elements present on the surface of IOMNPs. As can be seen in Fig. 4b, the XPS profile of O1s electrons can be decomposed in three peaks corresponding to oxygen in three different environments. According to the literature [45–47], they can be attributed as follows: 528.7 eV—oxygen in the Fe-O (lattice oxygen) component of magnetite; 530 eV—oxygen in an O-H, O-C, and O = C components; and 521.6 eV—oxygen in the bidentate bond of carboxylate moiety O-C = O. The deconvolution of C1s XP spectra (Fig. 4c) reveals the existence of three peaks which, according to the literature [44–47], can be assigned to the following: aliphatic or alkyl carbon at 285 eV, carbon single bonded to oxygen at 286.6 eV and carbon double bound to O or from carboxylate moiety (-O-C = O) at 288.6 eV, respectively. The XP spectra of the other samples (8 and 10 h), not shown here, are similar to those presented in Fig. 4.

While the nature of the most intense peak in the O1s XP spectra is rather clear (corresponding to the lattice oxygen of the magnetite), the origin of the chemical species that give rise to additional peaks in both O1s and C1s XPS spectra can be identified taking into account the possible reaction mechanisms that occurs in our system. Besides being used as a buffer, the acetate groups also stabilize the IOMNPs as they form. Therefore, the signals at 531.6 eV in O1s and 288.6 eV in C1s are most likely due to these acetate groups from the IOMNP surface. The hydrolysis of magnetic precursor FeCl_3_ (water is present in the reaction system as a part of the added salts, which are found only in the form of crystalohydrates) leads to the occurrence of Fe(OH)_3_ and HCl, which is neutralized by sodium acetate, resulting CH3COO- that binds to the surface of IOMNPS through the two oxygen atoms giving rise to the peaks at 532.1 and 288.2 eV in XP spectra of O1s and C1s electrons. Due to the high temperature, a fraction of Fe(OH)_3_ will dehydrate to form Fe_2_O_3_, while the rest of Fe(OH)_3_ will interact with PEG200, under the influence of which the Fe3^+^ ion is reduced to Fe2^+^. The C-C, C-O, and C = O components of physisorbed PEG200 and the C-H_3_ group of acetate attached to the IOMNP surface are identified in XP spectra by the O1s peak at 531.5 eV and C1s peaks at 285, 286.6, and 288.7 eV. For the samples prepared in 90 ml of PEG200, the Fe content at the surface (Additional file 1: Table S4) decreases while the amount of C increases compared with the samples prepared in 60 ml of PEG200. As a consequence, the C1s peak at 286.6 eV corresponding to C-O dominates the C1s XPS spectrum (Fig. 4i, j).

**Fig. 5:**
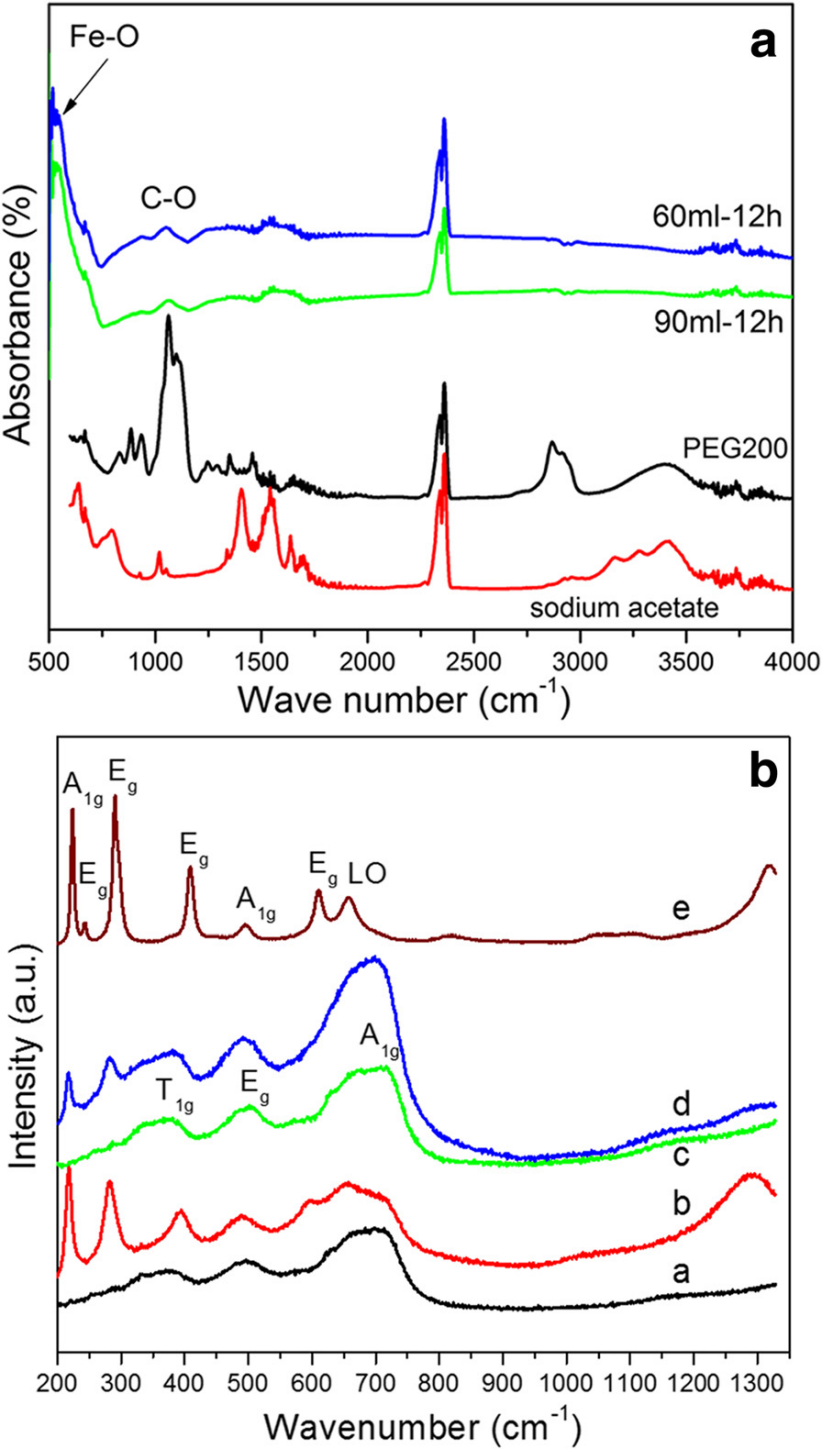
**a** FT-IR spectra of sodium acetate (*red*), PEG200 (*black*), and IOMNPs synthesized in 60 and 90 ml of PEG200 for 12 h (*green* and *blue*); **b** Raman spectra of IOMNPs powder and maghemite before (spectra *a* and *c*) and after local heating by the excitation laser (spectra *b* and *d*) and of hematite (spectrum *e*)

Further evidence for the presence of different molecular species on the surface of IOMNPs is obtained from FT-IR spectroscopy. Figure 5a shows the FT-IR spectra of IOMNPs obtained in 60 and 90 ml of PEG200 for a reaction time of 12 h together with the FT-IR spectra of PEG200 and sodium acetate. The FT-IR spectra of IOMNPs are dominated by a strong absorption band around 540 cm^−1^, which can be ascribed to Fe-O bond in Fe_3_O_4_ nanoparticles [27, 47], indicating that the main phase of the as-synthesized IOMNPs is Fe_3_O_4_. In 650–1450 cm^−1^ region, the FT-IR spectrum of PEG200 is characterized by several absorption bands (Fig. 5a). The most intense absorption band in this region, located at 1062 cm^−1^, attributed to vibration band of C-O bond, is the only absorption band of PEG200 which is faintly visible in the FT-IR spectra of IOMNPS (Fig. 5a). Similarly, the stretching vibrations of the carboxyl salt of the sodium acetate, located between 1300 and 1700 cm^−1^, are also feebly present in the FT-IR spectra of IOMNPs (Fig. 5a). These experimental observations suggest that very small amounts of PEG200 and acetate are present onto the surface of IOMNPs. These findings, combined with those obtained by XPS, support the assumption of a partial oxidation of IOMNPs surface.

In order to verify the oxidation degree of IOMNPs, we have analyzed the IOMNPs by means of Raman spectroscopy. This technique has emerged as a powerful tool for the investigation of oxide nanostructures, being capable to discern between different phases of IOMNPs [48]. As it can be seen in Fig. 5b (spectrum a), the Raman spectrum acquired on IOMNP powder exhibits peaks around 374, 497, and 708 cm^−1^. The relative positions of the peaks and the overall shape of the spectrum resemble to that of maghemite (spectrum c in Fig. 5b), which was obtained by annealing the magnetite powder at 250 °C for 4 h [48]. Note that, upon annealing, the color of the magnetic powder has changed from black to brown, which is characteristic to maghemite phase. The further annealing of the magnetite powder at 550 °C for 6 h induces the formation of hematite. The spectrum e in Fig. 5b clearly exhibits the characteristic vibrational modes of hematite [48]. Moreover, the transformation of magnetite into hematite can also be induced by the excitation laser of the Raman spectrometer [49]. Increasing the laser power to its maximum value (150 mW) and the integration time to 20 s leads to the occurrence of different peaks corresponding to hematite phase (spectrum b in Fig. 5b). For instance, the apparition of a second-order longitudinal phonon mode around 1300 cm^−1^ (spectrum b in Fig. 5b)—a feature characteristic to hematite phase (spectrum e in Fig. 5b) [49, 50]—confirms the conversion of magnetite intro hematite upon laser excitation. The same laser treatment applied to maghemite powder gives rise to the occurrence of two peaks of low intensity at small wavenumbers characteristic to hematite phases, whereas the three peaks of maghemite are better resolved (spectrum d in Fig. 5b). Since the maghemite phase is much more stable than magnetite, one can believe that a complete conversion into hematite will require higher energy. It can be thus concluded that IOMNPs are Fe_3_O_4_ whose surface is oxidized in maghemite.

### Magnetic Properties of IOMNPs

**Fig. 6:**
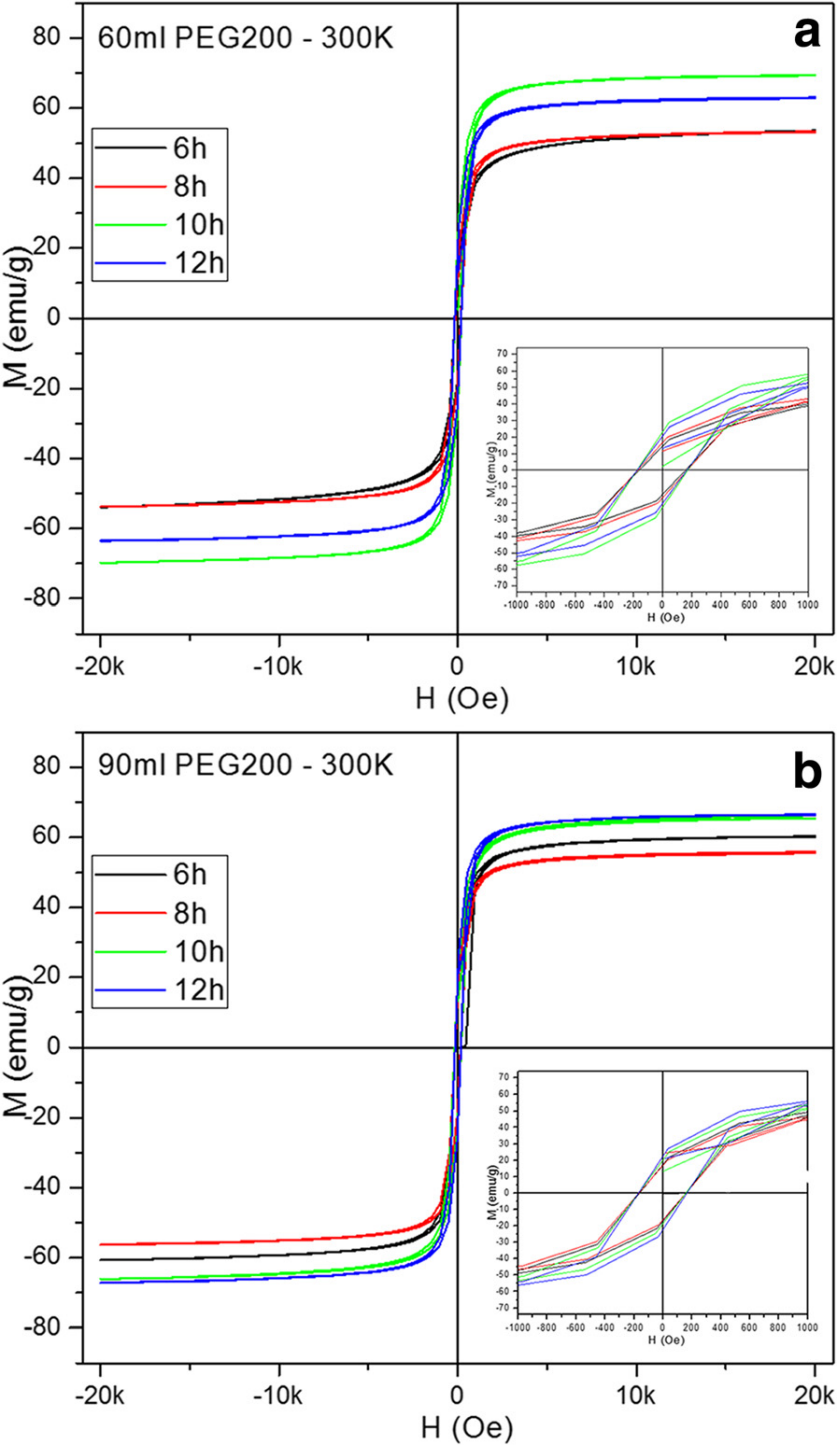
Magnetic hysteresis loops of IOMNPs obtained in **a** 60 and **b** 90 ml of PEG200 recorded at 300 K. The *insets* represent a zoom-in the hysteresis loops to evidence the coercive fields

The magnetic measurements performed on all samples indicate that the Fe_3_O_4_ MNPs are ferromagnetic at 4 K (Additional file 1: Figure S2). The IOMNPs exhibit saturation magnetization (*M*_*s*_) values starting with 62 emu/g and reaching 79 emu/g as the size of IOMNPs increases (Additional file 1: Table S5). The coercive field is on the order of 490–584 Oe (Additional file 1: Table S5), comparable with values reported for either cubic of spherical IOMNPs of smaller size [16, 48]. At 300 K, the IOMNPs preserve their ferromagnetic behavior (Fig. 5a, b), displaying coercive field of 170 and 160 Oe for IOMNPs synthesized in 60 or 90 ml of PEG200, respectively (Additional file 1: Table S6). The saturation magnetizations drop to values between 53.5 and 70 emu/g. These values are lower than the theoretical value of bulk magnetite (92 emu/g) and that of cubic IOMNPs with smaller dimensions synthesized by employing other techniques [22, 26, 48]. In principle, the significant decrease of the saturation magnetization can be due to several factors: the existence of different molecules adsorbed onto the surface of IOMNPs, the spin canting effect at the surface of IOMNPs, the formation of cation vacancies into the spinel crystalline structure of IOMNPs during synthesis, the oxidized surface of IOMNPs which can be regarded as a magnetically disordered shell surrounding the magnetic core. In our case, the first three factors can be discarded since the XRD patterns (Fig. 3) clearly indicate the formation of pure magnetic phases, the PEG and acetate molecules are only physisorbed onto the surface of IOMNPs (the IR spectra from Fig. 5a), while the spin canting is reduced for cubic magnetic nanoparticles [18]. On the other hand, it was previously reported that the oxidation of Fe_3_O_4_ NPs to maghemite induced a significant drop in the saturation magnetization from 83 to 70 emu/g [16]. Therefore, it seems reasonable to conclude that in our case, the oxidation of IOMNP surface is the main factor responsible for the lower values of the saturation magnetization at room temperature (Fig. 6).

In order to get more insights into the magnetic behavior of IOMNPs, the thermal dependence of the magnetization upon field cooling (FC) and zero field cooling (ZFC) has been recorded for all samples in a 50 Oe external field between 2 and 300 K. The maximum of ZFC curves, which corresponds to the average onset of the ferromagnetic to superparamagnetic transition, is located around 285 K for all samples (Additional file 1: Figure S3). At 300 K, all the IOMNPs are in the superparamagnetic state and the ZFC curves joins the FC ones. The broad peaks in ZFC curves (Additional file 1: Figure S3) at room temperature indicate a gradual transition to superparamagnetic state and suggest two aspects: strong magnetic interactions between IOMNPS and a granulometric distribution [51]. The FC curve behavior below the blocking temperature (Additional file 1: Figure S3) is an indication of the magnetic interactions established between IOMNPs [52]. The fact that for all the samples, the FC curves turn concave and then describe that a plateau below 100 K (Additional file 1: Figure S3) indicates that strong attractive magnetic interaction originated from magnetic dipole-dipole forces are manifested in between IOMNPs.

The M(T) values of FC curves can provide information about the inter-particle interaction strength [53]. A closer look to Additional file 1: Figure S3 reveal that the M(T) values of IOMNPs vary with the reaction time meaning that the dipole-dipole interaction strength changes with the shape and the size of IOMNPs. For IOMNPs synthesized in 60 ml of PEG200, the average M(T) values monotonously increase from 25 emu/g to 28 emu/for, 40 and 43 emu/g for 6-, 8-, 10-, and 12-h reaction time, respectively. In the case of IOMNPs synthesized in 90 ml of PEG200, the average M(T) values changes with the reaction time from a value of 37 emu/g for 6 h and reaches 43 emu/g for 12-h reaction time. It is thus expected a variation in the SAR values of IOMNPs with their size and shape. Given the fact that the remanent magnetization values are well below the 0.5 *M*_*s*_ (Additional file 1: Tables S5 and S6), the IOMNPs display an effective uniaxial anisotropy. Under ZFC conditions, the magnetizations display two transition temperatures characteristic to magnetite: one closed to 50 K, which can be attributed to a spin glass transition [54], and the second one around 100 K, corresponding probably to the Verwey transition temperature [48].

### Hyperthermia Properties of IOMNPs

As demonstrated above, the IOMNPs develop strong attractive inter-particle interactions that favor the formation of aggregates when they are dispersed in water. Taking into account that the heating efficiency of cubic magnetic nanoparticles is influenced by the arrangement in different patterns [53, 55–57], and for ensuring colloidal stability and hampering the formation of big clusters, 4 mg of each powder of IOMNPs have been dissolved in 1-ml aqueous solution of 25 % tetramethylammonium hydroxide (TMAOH), sonicated for 1 h and left over night. Subsequently, the IOMNPs have been magnetically separated and re-dispersed in 1-ml aqueous solution of 25 % TMAOH, following 30 min of sonication. Finally, the IOMNPs have been magnetically separated and re-dispersed in double distilled water. Upon this protocol, the TMAOH molecules attach to the IOMNP surface through the OH groups, thus preventing their further agglomeration into big cluster and sedimentation at the bottom of vial. However, as shown by the DLS measurements (Additional file 1: Figure S7, Table S7), IOMNPs are present as aggregates with hydrodynamic diameter smaller than 1 μm in aqueous solution. Hence, the heat performance of IOMNPs was measured in water at four different dilutions: 4, 2, 1, and 0.5 mg/ml. The SAR was measured for each sample at four different oscillating magnetic field strengths (10, 20, 40, and 60 kA/m) and two different frequencies (195 and 355 kHz). Before performing the set of SAR measurements at each magnetic field strength, the samples were sonicated for 30 s in order to assure a uniform distribution of the IOMNP nanoparticles in the sample volume. SAR values were calculated by measuring the initial slope of temperature vs. time curves and normalized to the IOMNP amount considering the heat capacity of water as briefly described in SI.

Heat release in a macroscopic sample of magnetic material is due only to magnetic hysteresis (the loop described by the magnetization of a sample as a function of the applied magnetic field). Owing to the fact that in electro-technique science the heating should be avoided and the heat released is considered a loss, the term hysteresis loss is also used. The energy loss during a cycle equals the area inside the hysteresis loop. This value multiplied by the frequency (number of cycles per unit time) gives the energy loss in unit time, i.e., the power loss.

For magnetic nanoparticles, other two processes leading to heat generation (losses) were described phenomenologically: the Neel relaxation and the Brown relaxation [58]. In the case of Neel relaxation, occurring in small, single domain particles, the macroscopic magnetization will tend to vanish after the external magnetic field is shut off due to the thermal agitation when thermal energy (*k*_*B*_*T*) surpasses the energy barrier needed to reverse the magnetization given by *KV*, where *V* is the volume of the nanoparticle and *K* is the magnetic anisotropy constant. The Brown relaxation is produced by the physical rotation of the MNP as a whole under the influence of the external magnetic field and the corresponding relaxation time is directly proportional to the viscosity of the suspending medium and to the hydrodynamic volume of the particle being inversely proportional to the thermal energy *k*_*B*_*T*. Although the hysteresis loss and the two relaxation phenomena are treated as distinct phenomena by many research groups, Carrey et al. [59] pointed out that the two relaxation phenomena could be treated within the frame of single dynamic hysteresis theory and that the relaxation processes should not be considered apart from the hysteresis losses. Depending on the MNP size, composition, coating, magnetic field frequency and amplitude, nature and viscosity of suspension medium, and temperature, the abovementioned phenomena contribute in different proportion to the overall heat released. Correctly quantifying the contribution of each heating mechanisms is mandatory for a proper design of the nanoparticles as a function of the application in which they are supposed to be used. Two main theoretical approaches were developed in this sense.

In the frame of the linear response theory (LRT), it is considered that the magnetization of the nanoparticles linearly depends on the applied magnetic field, the proportionality factor being the complex susceptibility. The rate of heat release in an alternating magnetic field can be written as [9]:

$$ P={\mu}_0\pi \chi^{{\prime\prime} }f{H}_{\max}^2 $$

where *μ*_*0*_ is the vacuum magnetic permeability, *f* the frequency, *H*_max_ the amplitude of the alternating magnetic field strength, and *χ* ″ the imaginary part of the magnetic susceptibility;

$$ {\chi}^{{\prime\prime} }={\chi}_0\frac{\omega \tau }{1+{\left(\omega \tau \right)}^2} $$

with the static susceptibility,

$$ {\chi}_0={\mu}_0\frac{M_s^2V}{a{k}_BT} $$

where *M*_*s*_ is the saturation magnetization of the material, *V* its volume, *a* = 3 for particles with *KV* < *k*_*B*_*T* (in this case the static Langevin susceptibility), and *a* = 1 if *KV* > *k*_*B*_*T. τ* is the relaxation time and in the case when both Brown and Neel relaxations occur,

$$ \frac{1}{\tau }=\frac{1}{\tau_N}+\frac{1}{\tau_B} $$

where *τ*_*N*_ and *τ*_*B*_ are the Neel and Brown relaxation times. The overall relaxation time will always be smaller than any of the two relaxation times, the smaller relaxation time dictating its value. For small MNP with diameter below a critical value *d*_*c*_, depending on the frequency of the alternating field and the viscosity of the medium, the Neel relaxation prevails [58] while for MNPs with larger diameters the Brown mechanism is dominant. The main result of the LRT is that the maximum power release is obtained when the angular frequency equals the reverse of the relaxation time *ωτ* = 1, and the imaginary part of the susceptibility presents a maximum. This maximum can be experimentally observed only for low size and monodispersed MNPs. For poly-dispersed samples, the superposition of different maxima for different sizes will mask the maximum; and consequently, the frequency dependence of the SAR is almost linear. Also, the LRT predicts a square dependence of the SAR on the amplitude of the magnetic field strength without any saturation effect. However, the LRT model is limited to small applied field strengths for which the magnetic energy is smaller than the thermal energy.

Another theoretical approach for calculating the SAR values was proposed by Stoner and Wohlfarth [60]. They considered the limit of anisotropic ellipsoids possessing only two orientations possible for the magnetization, without taking into account the thermal activation (*T* = 0). The magnetization can be reversed only by magnetic fields higher than a critical value *H*_*k*_. The hysteresis loop is rectangular and the coercive field equals the critical field and the anisotropy field. The area of the hysteresis loop is maximum and gives the upper limit of the SAR for a given material;

$$ \mathrm{S}\mathrm{A}\mathrm{R}=P/\rho =Af/\rho = 4{\mu}_0{M}_s{H}_cf/\rho $$

where *A* stands for the area of the hysteresis loop, *M*_*s*_ is the saturation magnetization, *H*_*c*_ is the coercive field, and *ρ* is the density. For example, for magnetite with *M*_*s*_ = 480 kA/m and *H*_*c*_ = 30 kA, the maximum achievable SAR at 500 kHz is 7 kW/g [58].

However in real cases of randomly oriented MNPs, the coercive field is reduced to 0.48 from the critical field and, as a consequence, the maximum SAR is reduced in the same field conditions to about 1/2 from the pure Stoner-Wohlfarth NPs [59]. For magnetic field amplitudes smaller than the coercive one, which are not able to reverse the magnetization of the MNPs, no energy absorption takes place. Therefore, this model holds true only at magnetic field amplitudes surpassing the coercive field, when the hysteresis loop is a major one and the MNPs are saturated by the magnetic field. Carrey et al. [59] showed that in the case of high amplitude magnetic fields, the coercive field can be obtained from hyperthermia experiments and its value can lead to the optimal size of MNPs maximizing the SAR. The *H*_*c*Hyp_ is calculated from the equation:

$$ {\mu}_0{H}_{c\mathrm{H}\mathrm{y}\mathrm{p}}=0.463{\mu}_0{H}_k\left\{1-{\left[\frac{k_BT}{KV} \ln \left(\frac{k_BT}{4{\mu}_0{H}_{c\mathrm{H}\mathrm{y}\mathrm{p}}{M}_sVf{\tau}_0}\right)\right]}^{0.8}\right\} $$

and represents the point of highest slope on the *SAR* = *f*(*H*) curve. The results obtained for the calculated coercive fields and the experimental SAR were in good agreement, the *SAR* = *f*(*H*) having a sigmoidal shape. As we will present below, our experimental *SAR* = *f*(*H*) also have a sigmoidal shape reaching saturation at about twice the coercive field.

In the case of an intermediate regime, none of the above models can be applied and numerical methods are usually employed, as it was proposed by the same group [59]. Even though this model does not provide accurate numerical data of the SAR it can correctly describe qualitatively the thermal properties of MNPs in alternating magnetic fields [61]. Christiansen et al. [62] improved the model in the frame of the dynamic hysteresis and introduced the multiplexing concept, i.e., the possibility to selectively heat MNPs with different magnetic properties and sizes by varying the characteristics of the magnetic field. They were able to show that at large magnetic field amplitudes and relatively low frequencies, large MNPs exhibit very high SAR values, which is again in accordance with our results.

**Fig. 7:**
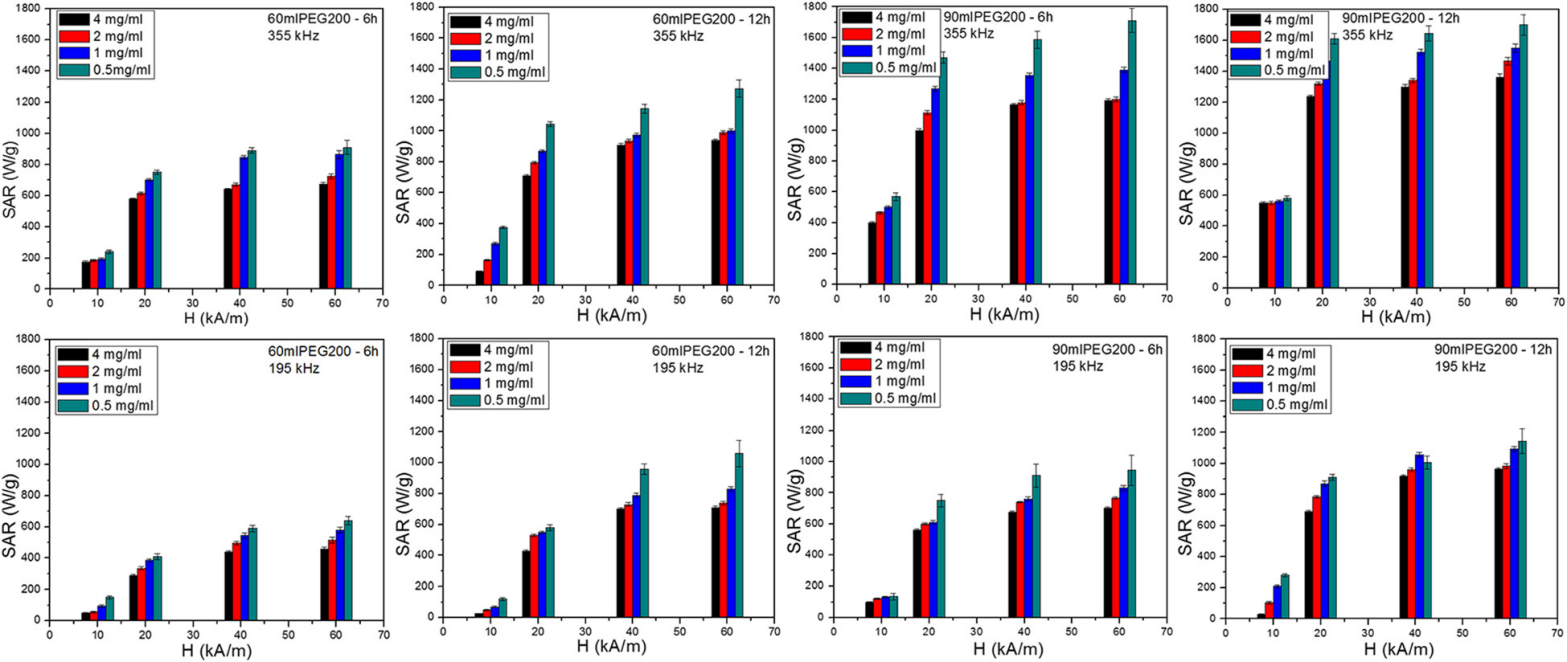
SAR values of IOMNPs synthesized in 60 and 90 ml of PEG200 for 6 and 12 h corresponding to four concentrations recorded at different applied magnetic fields and two frequencies: 355 kHz (*top panels*) and 195 kHz (*bottom panels*). The data are displayed as the mean of 5 measurements ± the standard error of the mean

**Fig. 8:**
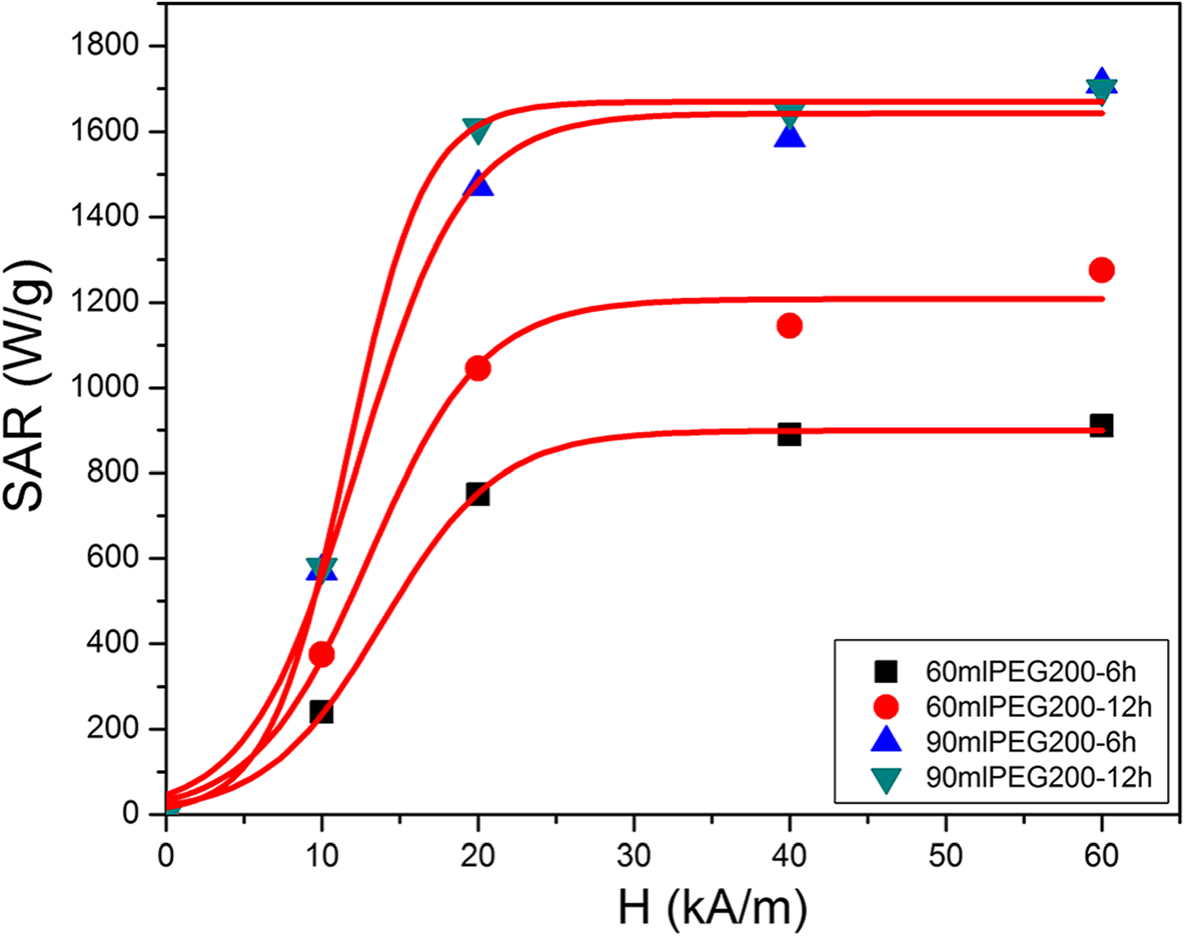
SAR values of IOMNPs synthesized in 60 and 90 ml of PEG200 for 6 and 12 h as a function of the applied magnetic field at 355 kHz and 0.5 mg/ml. The experimental results are fitted with a sigmoidal function

The resulting SAR values of the IOMNPs synthesized in 60 and 90 ml of PEG200 for 6 and 12 h, as a function of the applied magnetic field amplitudes at 195 and 355 kHz, are displayed in Fig. 7. As mentioned before, the SAR determination has been performed at magnetic field strength up to 60 kA/m, which is among the highest magnetic field ever reported in magnetic hyperthermia experiments. The use of this large magnetic field strength range allowed us to reach the saturation of the SAR values. Figure 8 exhibits the SAR values for all four samples as a function of applied magnetic field at a concentration of 0.5 mg/ml and at 355 kHz. The experimental data are very well fitted with a sigmoidal function. These results are in agreement with the numerical simulation performed by Carrey et al. [59] and Christiansen et al. [62]. Indeed for large ferromagnetic nanoparticles, the numerical simulation revealed an abrupt transition as a function of the magnetic field, from a regime where the hysteresis area is very small to a regime where the hysteresis area is very large. The explanation for this behavior is related to the coercive field (*H*_*c*_). When the applied field (*H*) is smaller than the *H*_*c*_, the hysteresis area is very small, while when *H* is larger than the *H*_*c*_, the hysteresis area is bigger. On the other hand, for very small magnetic fields, the SAR dependence can be described by a power law. In fact, in this domain, the LRT model is applicable and a parabolic dependence of SAR on the magnetic field was usually reported.

In our case, for an applied magnetic field amplitude of 10 kA/m (lower than the *H*_*c*_ of IOMNPs: 12.75 kA/m for 60 ml of PEG200 and 13.5 kA/m for 90 ml of PEG200), the IOMNPs, at a concentration of 0.5 mg/ml, display SAR values between 240 and 580 W/g at 355 kHz (Fig. 7). When the applied magnetic field amplitude (20 kA/m) overcomes the *H*_*c*_, the SAR values drastically increase (2.5–3 times) to 750–1610 W/g, since a larger hysteresis area is covered. The inflection points observed in the sigmoidal curves correspond to the *H*_*c*_ values obtained from magnetization curves (Fig. 8). According to the fits in Fig. 8, the SAR values reach saturation when the amplitude of the applied magnetic field is twice the value of *H*_*c*_, as pointed out by Carrey et al. [59]. A similar behavior of the SAR values as a function of the applied magnetic field was obtained at all concentrations and also at the frequency of 195 kHz (Figs. 7 and 8).

It can be noticed that SAR values increase almost directly proportional with the magnetic field frequency for all the samples at all the concentrations and amplitudes of the applied magnetic fields. This frequency dependence of the SAR is consistent with all theoretical models. However, for the highest values of the magnetic field strengths, there are slight deviations from the direct proportionality as the SAR values reach saturation. The absolute SAR values are very large, reaching 1700 W/g for both samples prepared in 90 ml of PEG200 at the highest frequency (355 kHz). These two samples provide the largest SAR values at 195 kHz as well. We were not able to obtain a clear-cut correlation of the maximum SAR values with the static magnetic properties of the MNP or to their size distribution revealed by the TEM data. However, the SAR data correlate with the plateau magnetization in the FC curves (Additional file 1: Figure S3). As the plateau magnetization is larger (the case of IOMNPs prepared in 90 ml of PEG200), dipole-dipole interactions are weaker and the magnetic heat dissipation is also larger. For the cubic IOMNPs synthesized in 60 ml of PEG200 at 6 h, the plateau magnetization is low, suggesting that these MNPs develop stronger dipole-dipole interactions and consequently, their SAR values are more affected. At both frequencies and for all four magnetic fields, the SAR values display the same trend: they increase as the IOMNP concentrations decreases [55]. This is a consequence of magnetic dipole-dipole attractive interactions acting between IOMNPs [55].

Many papers reported apparently contradictory data on the SAR dependence on the concentration of MNPs, some of them being summarized in [63] and [64]. In some studies, the decrease in SAR with an increase in MNP concentration was explained based on the dipole-dipole interaction leading to chain formation [64] and the subsequent SAR reduction. Deatsch and Evans [64] interpreted this effect by making a distinction between aggregation and agglomeration and also between the two relaxation mechanisms, Brown and Neel. Recently, Bianco-Andujar et al. [65] reported that the decrease in the heating performance of MNPs is due to the demagnetizing effect of the interparticle interactions and that multicore nanoparticle aggregates exhibit higher SAR values when larger core nanoparticles aggregate in smaller complexes than in the opposite situation.

In order to discern the contribution of different mechanisms to power dissipation, the IOMNPs were measured in more viscous solvent like PEG600. The SAR values (Additional file 1: Figure S4), for both type of IOMNPs for all applied magnetic fields, drastically decrease, indicating that the Brownian contribution is suppressed: the SAR values increase with the applied magnetic field amplitude reaching 600 W/g at 60 kA/m (355 kHz), where the highest hysteresis area is covered. A progressive decrease of SAR values is recorded when the viscosity is increased by dissolving the IOMNPs in PEG1000 (Additional file 1: Figure S4). Since the PEG1000 at room temperature is solid, the Brownian contribution is totally suppressed and the magnetic anisotropy losses contribute to the power dissipation. Therefore, one can consider that Brownian relaxation is the main mechanism responsible for the high SAR values obtained in water for both type of IOMNPs. The dipole-dipole attractive interactions induce aggregation of IOMNPs. As depicted in the Additional file 1: Figure S7 in DLS measurements, the IOMNPs exhibit different degrees of aggregation. At a concentration of 0.5 mg/ml, cubic IOMNPs form aggregates with hydrodynamic diameters between 450 and 620 nm, while polyhedral IOMNPs of smaller size develop aggregates with bigger hydrodynamic diameters (700–850 nm). It can be observed that the SAR values of IOMNPs increase with the hydrodynamic diameter of IOMNP aggregates (Additional file 1: Figure S7). This observation is consistent with the above presented results proving that Brown relaxation is the main mechanism for heat generation in large IOMNPs at high magnetic field intensities.

Several studies in the literature have reported outstanding SAR values for cubic IOMNPs [22, 26, 55, 57]. For instance, around a *H* × *f* factor of 7 × 10^9^ A/m × s, non-interacting monodispersed cubic IOMNPs of 19 and 35 nm exhibit SAR values of 1000 and 1391 W/g_Fe_, respectively [22, 26], whereas 23 nm cubic IOMNPs aggregated inside 200-nm magnetic nano-beads display a SAR values of 194 W/g_Fe_ [57]. Furthermore, a few hundreds of watt per gram are reported for strongly interacting cubic IOMNPs with size of 20 or 40 nm [55]. Therefore, the SAR values reported herein are comparable with the non-interacting monodispersed cubic IOMNOPs and are higher than the aggregated ones. Thus, these SAR values confirm that the IOMNPs could be excellent heat mediators for hyperthermia treatment. Obviously, the high SAR values displayed by our IOMNPs are mainly given by the Brownian friction within the water which increases with the hydrodynamic volume of IOMNPs and of aggregates. However, for a proper use of these IOMNPs in biological applications, the synthesis method should be improved, in order to reduce the IOMNP polydispersity as well as the surface oxidation.

In the last years, the vast majority of magnetic hyperthermia studies were performed on ultra-small single-domain MNPs as they were considered the best suitable for the use in clinical hyperthermia applications, due to their small sizes and superparamagnetic state. However, the most important characteristic of the MNPs for their use in such applications is their capability to heat human tissues when they are exposed to an alternating magnetic field. For the very small IOMNPs, the heat release rate is limited. As pointed out recently by Kashevsky et al. [66], hard larger MNPs, with pronounced stationary hysteresis much closer to the Stoner Wohlfarth model, are theoretically able to release 24 times larger heat as compared to the small superparamagnetic ones and should be considered as good candidates for human hyperthermia applications. The major drawbacks in such applications for the large MNPs is related to their possible agglomeration and aggregation in vivo together with lower SAR values due to suppression of the Brownian relaxation by their immobilization either to the cell membranes or in the cytoplasm. Contrary to these opinions, Dennis et al. [67] reported that large dextran-coated IOMNPs are able to release in vivo enough heat to induce complete tumor regression in mice.

## Conclusions

IOMNPs have been successfully synthesized by thermal decomposition of FeCl_3_ in the presence of PEG200 and sodium acetate, in a solvothermal system. This approach allows the synthesis of poly-dispersed cubic and polyhedral IOMNPs displaying high crystallinity and broad sizes, which can be tuned between 30 and 230 nm by varying the reaction time and the PEG200 amount. Sixty milliliters of PEG200 induce the formation of cubic IOMNPs and favor oxidative conditions which allow the formation of a small percent of hematite. An increased volume of PEG200 (90 ml) develops cubic and polyhedral IOMNPS and prevent the oxidation of magnetite into hematite. XPS analysis of IOMNPs clearly indicates that IOMNPs comprise a crystalline magnetite core, which undergoes oxidation and the presence of functional groups of PEG200 and acetate on the IOMNPs surface, while in the FT-IR spectra, the physisorbed PEG200 molecules are faintly visible. We demonstrated that RAMAN spectroscopy is a powerful technique able to monitor the oxidation state at the surface of the MNPs. The magnetic characterization performed at room temperature on powder reveal that IOMNPs are ferromagnetic with a coercive field of 160–170 Oe. The evolution of magnetization with the temperature in FC curves below the blocking temperature (300 K) indicates the occurrence of strong magnetic dipole-dipole interactions between IOMNPs, which vary with both shape and size. Consequently, the SAR values of IOMNPs, obtained in four different magnetic fields at two frequencies, depend on the concentration of IOMNPs in water. The SAR values of IOMNPs significantly increase for applied magnetic field exceeding the coercive field, since larger hysteresis loops are covered. From the fitting of SAR values as a function of applied magnetic field with a sigmoidal function, it has been found that SAR values reach saturation when the amplitude of the applied magnetic field is twice the coercive field. At a frequency of 355 kHz, the cubic and polyhedral IOMNPs of lower size prepared in 90 ml of PEG200 release higher amounts of heat (up to 1700 W/g) than the large cubic IOMNPs synthesized in 60 ml of PEG200 (1275 W/g). These large SAR values arise mainly from Brownian frictions of IOMNPs in water. Consequently, we believe that the here reported large IOMNPs have an unexplored potential for hyperthermia.

## Additional File

Additional file 1:
**It contains a TEM image of IOMNPs, the magnetic hysteresis at 4 K of IOMNPs, the ZFC-FC curves of IOMNPs, the SAR values of IOMNPs in PEG600 and PEG1000, DLS spectra of IOMNPs, and information related to the XRD and XPS analysis and magnetic properties of IOMNPs.** The calibration of the hyperthermia setup and the experimental and theoretical protocols for SAR determination are fully described as well. (DOCX 1448 kb)

## Abbreviations

FC: Field cooled
FT-IR: Fourier transform infrared spectroscopy
IOMNPs: Iron oxide magnetic nanoparticles
LRT: Linear response theory
MNPs: Magnetic nanoparticles
NaAc: Sodium acetate
NPs: Nanoparticles
PEG: Polyethylene glycol
SAR: Specific absorption rate
SPL: Specific power loss
T_B_: Blocking temperature
TEM: Transmission electron microscopy
TMAOH: Tetramethylammonium hydroxide
XPS: X-ray photoemission spectroscopy
XRD: X-ray diffraction
ZFC: Zero field cooled
