# Supplementary material for: Polyethylene Glycol-Mediated Synthesis of Cubic Iron Oxide Nanoparticles with High Heating Power
Source: Nanoscale Res Lett. 2015 Oct 7;10:391. doi: 10.1186/s11671-015-1091-0 (PMC4596149; doi:10.1186/s11671-015-1091-0)
Supplement: Additional file 1: — It contains a TEM image of IOMNPs, the magnetic hysteresis at 4 K of IOMNPs, the ZFC-FC curves of IOMNPs, the SAR values of IOMNPs in PEG600 and PEG1000, DLS spectra of IOMNPs, and information related to the XRD and XPS analysis and magnetic properties of IOMNPs. The calibration of the hyperthermia setup and the experimental and theoretical protocols for SAR determination are fully described as well. (DOCX 1448 kb) [file 11671_2015_1091_MOESM1_ESM.docx]

Supplementary Information

Polyethylene glycol mediated synthesis of cubic iron oxide nanoparticles with high heating power

Cristian Iacovita,*^a^* Rares Stiufiuc,*^a*^* Teodora Radu,*^b,c^* Adrian Florea,*^d^* Gabriela Stiufiuc,*^e^* Alina Dutu,*^f^* Sever Mican,*^e^* Romulus Tetean,*^e^* and Constantin M. Lucaciu *^a*^*

Figure S1: (a) TEM image of cubic IOMNPs obtained in 60ml of PEG200 for 6h by increasing the temperarure at a rate of 5˚C/minute and (b) its corespoding size distribution histrograms fitted to a log-normal distribution (black line).


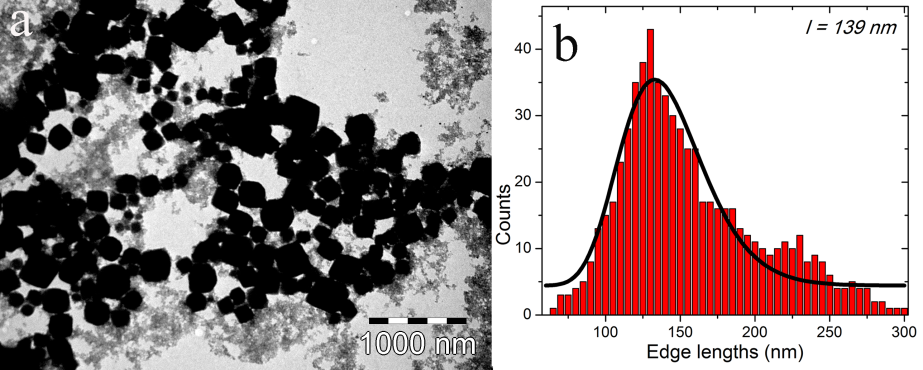


Table S1: Lattice parameters and the percentage concentration of both crystalline phases corresponding to IOMNPs synthesized in 60ml PEG200.

| Sample | Fe_3_O_4_ | | Fe_2_O_3_ | | |
| --- | --- | --- | --- | --- | --- |
|  | a (Å) | c (wt%) ± 2% | a (Å) | c (Å) | c (wt%) ± 2% |
| 60ml-6h | 8.37 | 94 | 5.04 | 13.75 | 6 |
| 60ml-8h | 8.38 | 95 | 5.04 | 13.76 | 5 |
| 60ml-10h | 8.38 | 97 | 5.04 | 13.74 | 3 |
| 60ml-12h | 8.38 | 93 | 5.04 | 13.76 | 7 |

Table S2: Lattice parameters of single crystalline phase corresponding to IOMNPs synthesized in 90ml PEG200.

| Sample | Fe_3_O_4_ |
| --- | --- |
|  | a (Å) |
| 90ml-6h | 8.38 |
| 90ml-8h | 8.37 |
| 90ml-10h | 8.39 |
| 90ml-12h | 8.37 |

Table S3: Percentage atomic content of iron species as determined from XPS survey spectra of the investigated samples surface.

| Sample | Fe^2+^oct (%) | Fe^3+^oct (%) | Fe^3+^tet (%) | Fe^2+^/Fe^3+^ |
| --- | --- | --- | --- | --- |
| 60ml-6h | 12.8 | 15.4 | 11.6 | 0.47 |
| 60ml-12h | 10.7 | 14.4 | 8.7 | 0.46 |
| 90ml-6h | 16.35 | 14.3 | 20.4 | 0.47 |
| 90ml-12h | 19.8 | 14.2 | 17.8 | 0.61 |

Table S4: Percentage atomic content of the elements as determined from XPS survey spectra of the investigated samples surface.

| Sample | C (at%) | O (at%) | Fe (at%) |
| --- | --- | --- | --- |
| 60ml-6h | 21 | 50.3 | 28 |
| 60ml-12h | 25 | 51 | 23.6 |
| 90ml-6h | 34 | 49.8 | 16 |
| 90ml-12h | 32.2 | 50.2 | 17.5 |

Figure S2: Magnetic hysteresis loops of IOMNPs recorded at 4K.


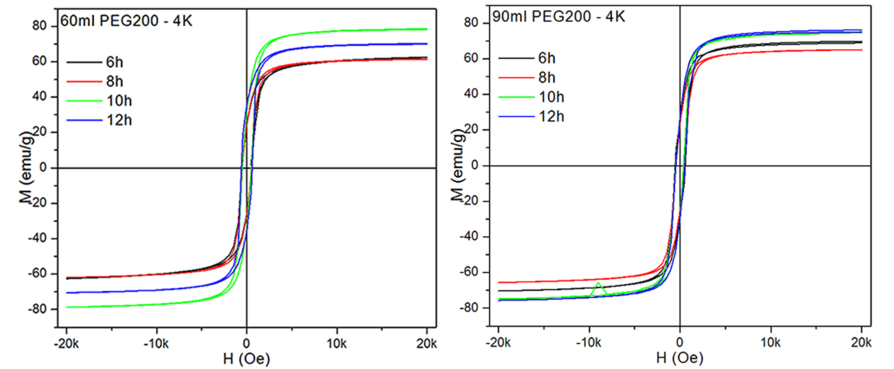


Table S5: Coercitive field, remanent magnetization and saturation magnetization of IOMNPs at 4K.

| Sample | H_c_  (Oe) | M_r_  (emu/g) | M_s_  (emu/g) |
| --- | --- | --- | --- |
| 60ml-6h | 514 | 24,5 | 62,5 |
| 60ml-8h | 514 | 24,5 | 62 |
| 60ml-10h | 514 | 32,5 | 79 |
| 60ml-12h | 584 | 36,5 | 70,5 |
|  |  |  |  |
| 90ml-6h | 562 | 26 | 70,5 |
| 90ml-8h | 490 | 24,5 | 65,5 |
| 90ml-10h | 490 | 29 | 75 |
| 90ml-12h | 490 | 29 | 76 |

Table S6: Coercitive field, remanent magnetization and saturation magnetization of IOMNPs at 300K.

| Sample | H_c_  (Oe) | M_r_  (emu/g) | M_s_  (emu/g) |
| --- | --- | --- | --- |
| 60ml-6h | 170 | 14,5 | 54 |
| 60ml-8h | 170 | 17 | 53,5 |
| 60ml-10h | 170 | 22 | 70 |
| 60ml-12h | 170 | 20 | 63,5 |
|  |  |  |  |
| 90ml-6h | 160 | 16,5 | 60,5 |
| 90ml-8h | 160 | 16,5 | 56,5 |
| 90ml-10h | 160 | 18,5 | 66 |
| 90ml-12h | 160 | 23 | 67 |

Figure S3: ZFC-FC curves of IOMNPs synthesized in 60mlPEG 200 (top panels) and in 90mlPEG200 (bottom panels).


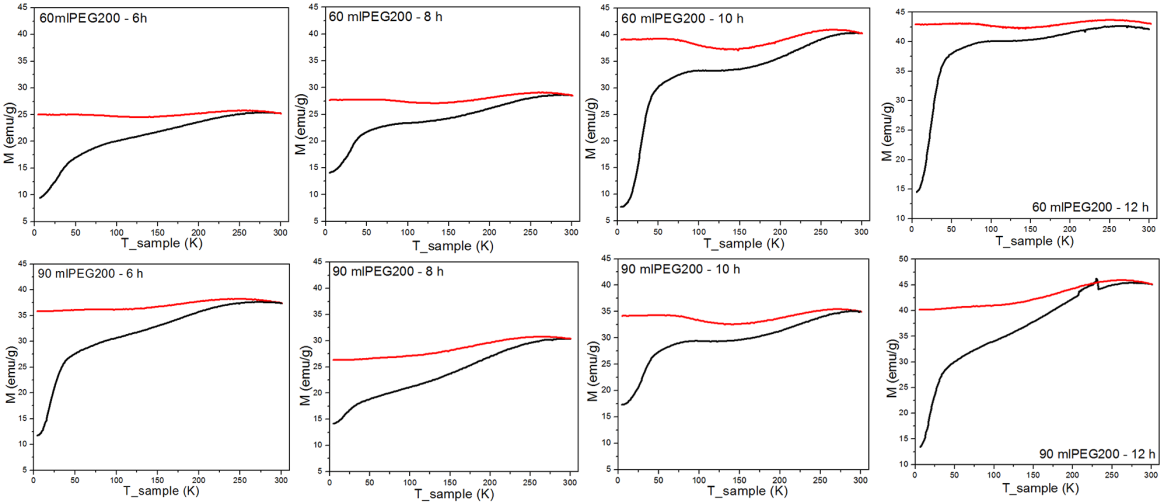


Figure S4: SAR values of IOMNPs synthesized in 60ml and 90mlPEG200 for 12h dispersed in PEG600 (left and middle panels) and PEG1000 (right panel) recorded at 355 kHz in different applied magnetic fields. The data are displayed as the mean of 5 measurements ± the standard error of the mean.


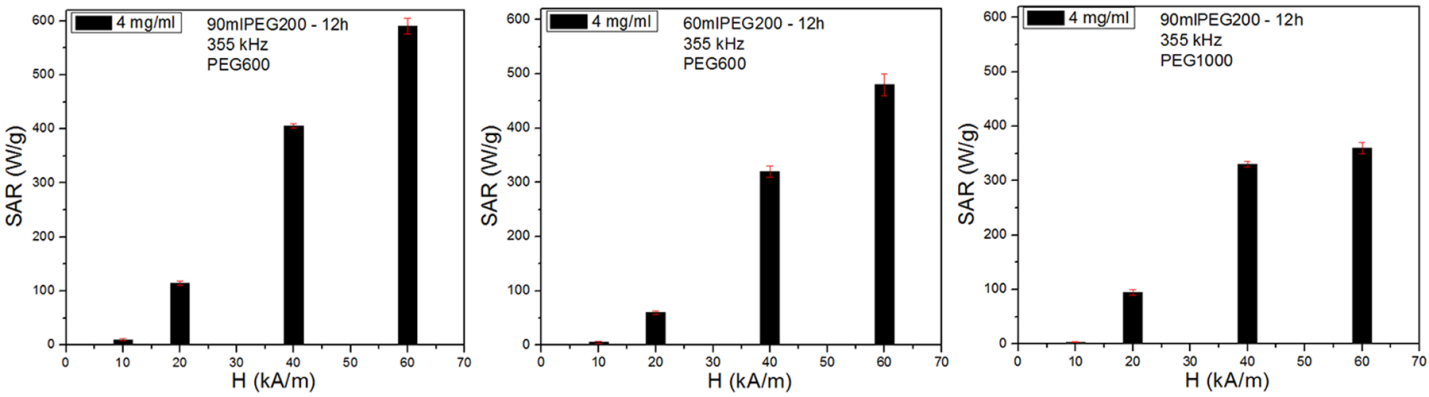


**Magnetic field strength calibration**

For characterizing the magneto-caloric properties of the synthesized nanoparticles we used an EasyHeat 0224 power supply (Ambrell, Scottsville, NY, USA), operating at frequencies between about 100-400 kHz and able to generate magnetic fields with strengths of up to 65 kA/m. The system is equipped with several coils made ​​of copper tubes that circulate water for cooling. We used a 8 turns coil with an internal diameter of 2.5 mm and a total length of 40 mm (figure S5). The inductance of the coil was calculated from both its geometry and the resonance frequency of the circuit in which it was introduced and was determined as 10^-6^ H. The effective frequency and voltage values on the coil ​​were monitored with a digital oscilloscope PeakTech 1170 operating up to 250 MHz.

In order to calibrate the magnetic field strength inside the solenoid coil we placed a single circular loop copper wire with 10 mm diameter, surrounding the vial in which the samples were introduced. The loop was placed centered on the symmetry axis of the solenoid and normal to it, in the middle and we measured the induced electromotive force by using the oscilloscope. Due to the modulation of the signal, the amplitude of induced electromotive force was calculated as the mean value of about 30 sinusoids. From the well-known Faraday’s induction law we derived the magnetic flux density values B, normal to the surface of the loop:

$$e=-\frac{\partial\Phi}{\partial t}=-\frac{\partial}{\partial t}\iint_{circle} \boldsymbol{BdS=}=-\frac{\partial}{\partial t}\iint_{circle} B_{n}dS\boldsymbol{=}-\omega B_{nmax}\pi r^{2}cos\omega t$$

where B_nmax_ = μ_0_H_nmax_ represents the magnetic field density amplitude mediated over the surface area of the loop and is used to calculate the magnetic field strength's amplitude. r and ω represent the radius of the circular loop and the pulsation of the field, respectively. Due to the high axial symmetry of the field lines the radial component of the field was neglected. The magnetic density varies along the z axis of the solenoid according to the formula [M. Zahn, Electromagnetic Field Theory: A Problem Solving Approach, Krieger Publishing Company, 2003]:

$$H=\frac{NI}{2L}\left[ \frac{-z+\frac{L}{2}}{\sqrt{\left( -z+\frac{L}{2} \right)^{2}+R^{2}}}+\frac{z+\frac{L}{2}}{\sqrt{\left( z+\frac{L}{2} \right)^{2}+R^{2}}} \right]$$

where N is the number of turns, I is the electric current intensity, L is the length of the solenoid, R is the solenoid radius and z is the distance from the center of the solenoid to the point on the axis where the magnetic field strength is calculated. The calculated magnetic field strength values obtained using this formula were compared to the experimental ones obtained by moving stepwise the loop along the z axis of the solenoid and measuring the electromotive force and are represented in figure S5.

Figure S5. Left-the solenoid used for hyperthermia measurements; Middle - experimental and theoretical values of the magnetic field strength along the z axis of the solenoid; Right – a sketch of the tube containing 0,5 ml aqueous solution of IOMNPs and its position inside the solenoid – the fiber-optic thermocouple is placed in the middle of the volume occupied by the aqueous solution of IOMNPs.


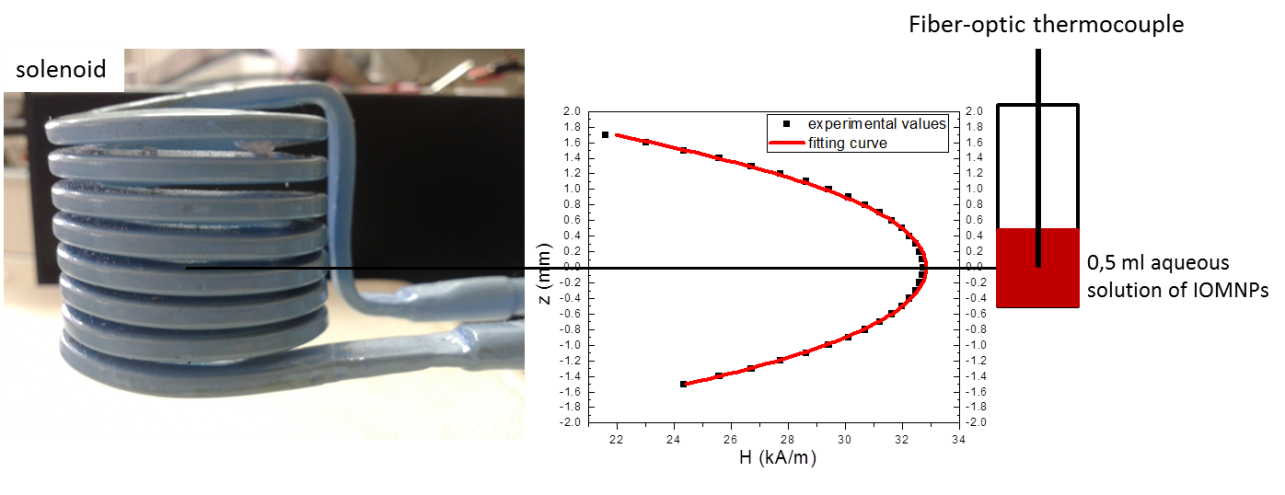


The calibration was performed at both frequencies used in this study. Based on this axial variation of the magnetic field strength we calculated its mean value 5 mm bellow and 5 mm above the center of the solenoid (the upper and lower limits of the MNP suspensions within the testing vials) by numerical integrating the fitting curve and we obtained a mean value for the magnetic field strength which is 98.6% from the its maximum in the center of the solenoid. These values ​​were used to calculate further the magnetic field strengths values for different electric current intensity amplitudes in the coil.

**Measurements of the Specific Absorption Rates (SAR)**

From the experimental point of view, most authors characterize magnetic nanoparticles through the so-called specific absorption rate (SAR), called sometimes specific power loss SLP, defined as the heat released from a suspension of nanoparticles in unit time reported to their mass, expressed usually in W/g:

$$SAR=\frac{P}{m_{MNP}}=\frac{Q}{\Delta t m_{MNP}}$$

the heat being calculated as the equivalent heat needed to increase the sample temperature with the same number of K in a given time interval,

$$Q=mc\Delta T$$

where Q is the heat, m is the mass of the sample subjected to is heating in alternating magnetic field, c specific heat of the sample (with a good approximation one can consider specific heat of water for dilute magnetic nanoparticles suspended in water), ΔT is the temperature increase of the sample, and Δt is the time during which the alternating magnetic field was applied.

The magnetic nanoparticles were weighed and then were suspended in double distilled water to the desired concentrations (4mg/ml, 2mg/ml, 1mg/ml and 0.5 mg/ml) for measurements of their hyperthermic properties. An amount of 0.5 ml of each sample was placed in round bottom test tubes with a total 2 ml capacity which was then positioned in the center of the coil (figure S5), surrounded by a special heat insulating support. Care was taken for the samples to be placed in the same position each time inside the coil to be subject to the same field strengths.

The temperature was measured with an optical fiber thermocouple coupled to a computer which allows temperature measurement every second. Note that fiber optics thermocouple provides precise temperature measurement without being influenced by the magnetic field. Every time the optical fiber thermocouple was placed in the middle of the volume occupied by the aqueous solution of IOMNPs (figure S5). It was found that in case of placing the thermocouple on the bottom of the volume the time dependence of the temperature is nonlinear, with a variable slope that decreases over time. If the thermocouple was placed in the center of the volume, the time dependence of the temperature is linear at least the first few tens of seconds and provided that the sample is not heated at temperatures with more than 10K higher than the temperatures of the environment, these observations being consistent with those made recently by Wildeboer *et al.* (Wildeboer R. R., Southern P., Pankhurst Q. A., On the reliable measurement of specific absorption rates and parameters intrinsic loss in magnetic materials, Journal of Physics D: Applied Physics 47, 495003, 2014).

For these reasons we preferred to use shorter measurement time of several tens of seconds, and to take into account only the linear portion of the time dependence of the temperature (figure S6) instead of using the Box-Lucas equation for fitting. The linear interpolation was applied for the calculation of the slope dT/dt. For all samples the linear regression coefficient R2 was higher than 0.999. All samples were measured at least 5 times and the samples were sonicated for 30 seconds before the measurement in order to ensure uniform dispersion of the particles in the suspension medium.

Figure S6. Registered typical temperatures changes for a suspension of nanoparticles subjected to an alternating magnetic field.

Figure S7. (a) Hydrodynamic diameter of IOMNPs (0.5 mg/ml) after treatment with TMAOH. (b) SAR values of IOMNPs (0.5 mg/ml; 355 kHz, 60kA/m) as a function of hydrodynamic diameter.


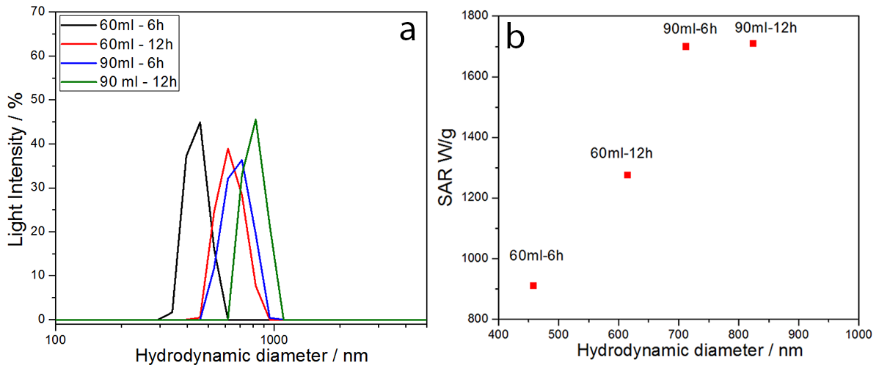


Table S7. Hydrodynamic diameter from DLS and mean size from TEM of IOMNPs.

| Sample | Hydrodynamic size (nm) | Size from TEM (nm) |
| --- | --- | --- |
| 60 ml PEG200 – 6h | 458 | 128 |
| 60 ml PEG200 – 12h | 615 | 230 |
| 90 ml PEG200 – 6h | 712 | 30 |
| 90 ml PEG200 – 12h | 824 | 48 |
